# Prognostic value of C-reactive protein to albumin ratio in metastatic colorectal cancer: A systematic review and meta-analysis

**DOI:** 10.1097/MD.0000000000027783

**Published:** 2021-11-19

**Authors:** Yan Pan, Yinmei Lou, Lin Wang

**Affiliations:** Department of Integrative Oncology, First People's Hospital of Fuyang, Hangzhou, Zhejiang Province, China.

**Keywords:** C-reactive protein to albumin ratio, meta-analysis, metastatic colorectal cancer, prognosis

## Abstract

**Background:**

In recent years, several observational studies have investigated the association between C-reactive protein to albumin ratio (CAR) and prognosis of metastatic colorectal cancer (mCRC), and yielded controversial outcomes.

**Methods:**

Eligible studies assessing the relationship of CAR with survival and clinicopathological parameters in mCRC were searched from PubMed, Cochrane library, and Embase databases up to February 3, 2021. Overall survival (OS), progression-free survival, recurrence-free survival, and disease-free survival were synthetically calculated and compared.

**Results:**

A total of 6 studies including 771 patients were enrolled in this systematic review. Pooled results indicated that elevated CAR was significantly associated with poorer OS (hazard ratio: 2.393; 95% confidence interval: 1.949–2.938, *P* < .01) as well as decreased progression-free survival/disease-free survival/recurrence-free survival (hazard ratio: 1.731; 95% confidence interval: 1.261–2.375, *P* < .01). Additionally, high CAR was significantly consistent with increased modified Glasgow Prognostic Score and neutrophil–lymphocyte ratio.

**Conclusion:**

High CAR could be a negative prognostic marker for mCRC patients. More large-sample clinical trials are still needed to confirm the prognostic significance of CAR in mCRC.

## 1 Introduction

Colorectal cancer (CRC) is one of the most common malignancies expected to be diagnosed worldwide. In 2020, an estimated 147,950 new CRC cases and about 53,200 cancer-related deaths are projected to occur in the US.^[1]^ Stagnant survival outcomes for CRC are largely attributed to limited progress in clinical treatment for cancer recurrence and metastasis.^[2]^ The prognosis of metastatic colorectal cancer (mCRC) remains even worse, with a 5-year survival rate of approximately 11%.^[3]^ Therefore, identification and application of ideal prognostic markers is crucial for advancement of diagnosis and prognosis in mCRC patients.

It is widely recognized that inflammation plays an important role in cancer development and progression.^[4–6]^ Inflammation stimulates the release of cytokines, such as cytokine interleukin 6 (IL-6), tumor necrosis factor-α (TNF-α) etc, and consequently inhibit cell apoptosis and promote DNA damage, which contribute to tumor growth, invasion and metastasis.^[7,8]^ Notably, some inflammation-based scoring system, such as platelet–lymphocyte ratio and neutrophil–lymphocyte ratio (NLR), have been in-depth investigated in mCRC as prognostic indicators.^[9,10]^ As a novel inflammatory scoring index, the C-reactive protein to albumin ratio (CAR) has been reported recently to be a reliable marker in predicting survival of mCRC.^[11–16]^ Haruki et al^[14]^ and Sakamoto et al^[16]^ found that patients with elevated CAR had poorer OS, disease-free survival (DFS)/recurrence-free survival (RFS). Nevertheless, Shibutani et al^[15]^ got opposite results of the relationship between high CAR and progression-free survival (PFS). In addition, the sample size of these studies was small, and the results were conflicting. Thence, we conducted the current meta-analysis to comprehensively and quantitatively evaluate the association of CAR with survival outcomes and clinicopathological parameters based on available mCRC related literatures.

## 2 Materials and methods

This meta-analysis was performed following preferred reporting items for systematic reviews and meta-analysis criteria.^[17]^ The ethical approval was not required for this study because no data of individual patient information was used. Systematic review registration was finished online and presented as the International Platform of Registered Systematic Review and Meta-Analysis Protocols (INPLASY) 202120013.

### 2.1 Search strategy

**Figure 1:**
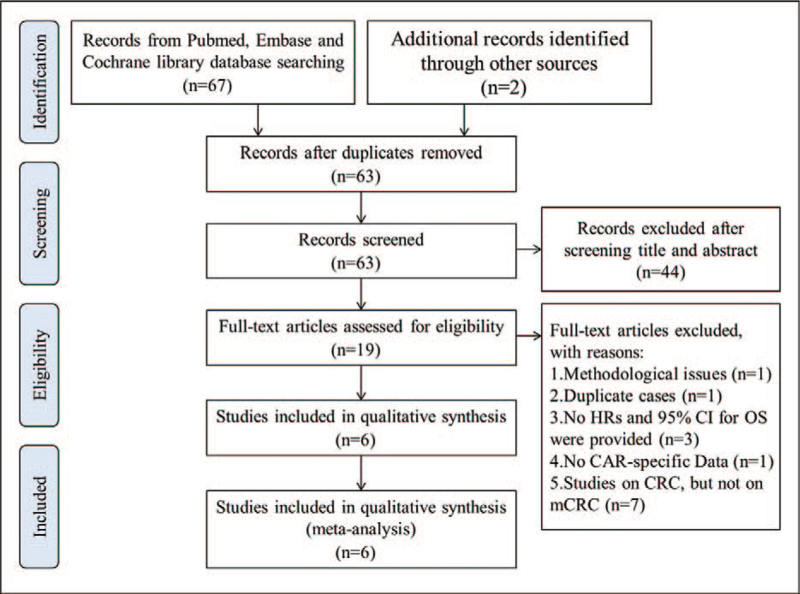
Flow diagram of literature selection process for this meta-analysis. CAR = C-reactive protein to albumin ratio, CI = confidence interval, CRC = colorectal cancer, HR = hazard ratio, mCRC = metastatic colorectal cancer, OS = overall survival.

Two investigators independently searched PubMed, Cochrane library, and Embase databases up to February 3, 2021 for eligible studies. The following search terms were used: (“C-reactive protein to albumin ratio” or “C-reactive protein/albumin ratio” or “C-reactive protein albumin ratio” or “C-reactive protein-to-albumin ratio”) and (“carcinoma” or “neoplasm” or “tumor” or “cancer”) and (“colorectal” or “rectal” or “colon”) and (“survival” or “death” or “mortality”) and (“metastases” or “metastatic”). Reference lists of included articles were manually checked for extra data (Fig. 1).

### 2.2 Inclusion and exclusion criteria

The following inclusion criteria were used for selecting the eligible studies: patients with mCRC were pathologically confirmed; CAR was measured by serum based methods; correlation of CAR with clinical outcomes including overall survival (OS), DFS, PFS, or RFS; and study type of article should be cohort study.

Exclusion criteria were as follows: abstracts, letters, case reports, reviews, meta-analysis or nonclinical studies; studies with insufficient data for estimating hazard ratios (HRs) and 95% confidence intervals (CIs); studies had duplicate data or repeat analysis; and full text cannot be obtained.

### 2.3 Data extraction and quality assessment

All eligible studies were independently reviewed and evaluated by 2 authors (YP and LW). The following information was retrieved: first author, year of publication, country, sample size, study design, cutoff value of CAR, age, gender, follow-up, treatment, tumor location, detection of metastatic tumor, differentiation, no. of organs affected by metastasis, carcinoembryonic antigen (CEA), serum lactate dehydrogenase (LDH) level, modified Glasgow Prognostic Score (mGPS) and NLR, survival outcomes (OS, DFS, PFS, RFS). Methodological quality assessment of candidate studies was appraised on the basis of Newcastle–Ottawa Quality Assessment Scale (NOS).^[18]^ Studies with NOS score of 7 points or higher are considered as high-quality articles. Disagreements in data extraction and quality assessment were resolved by consensus.

### 2.4 Statistical analysis

Analyses were conducted with STATA version 16.0 (Stata Corp, College station, TX). HRs and 95% CIs were directly extracted from each included researches to pool the prognostic value of CAR for OS and other clinical outcomes such as PFS, DFS, RFS, and odds ratio (ORs) were calculated to find the association between high CAR and clinicopathological features. The heterogeneity of the eligible articles was evaluated by Cochrane Q test and *I*^2^ statistics. *P* < .1 or *I*^2^ > 50% indicated significant heterogeneity. The random-effects model was applied to analyze the pooled HRs or ORs which had significant heterogeneity; otherwise, the fixed-effects model was used. Subgroup analysis was performed to explicit the heterogeneity among all eligible studies base on country (Japan, China or UK), cutoff value of CAR (≥0.1 or <0.1), therapy (chemotherapy, surgery or mixed), sample size (≥150 or <150), NOS score (≥7 or <7), and analysis (univariate or multivariate). Publication bias was estimated using Begg,^[19]^ Egger^[20]^ and the trim and fill method.^[21]^ Sensitivity analysis was conducted to validate the stability of the pooled results by omitting each study.

## 3 Results

### 3.1 Study selection

A total of 69 relevant studies were retrieved through literature searching of Pubmed, Cochrane Library, and Embase databases. Among these articles, 6 duplicates were removed, and 19 studies remained for further assessment after reviewing the titles and abstracts. After full-text screening, 13 articles were excluded according to the inclusion criteria. Six eligible studies involving 771 patients were finally enrolled in this meta-analysis.^[11–16]^ The literature selection process was shown in Figure 1.

### 3.2 Study characteristics

The main characteristics of the eligible articles are summarized in Table 1. Among these retrospective cohort studies published between 2016 and 2020, 4 programs^[11,14–16]^ were conducted in Japan and 1 each in United Kingdom^[13]^ and China,^[12]^ and the sample sizes ranged from 40 to 194. Three studies^[11,12,15]^ compromised patients with chemotherapy, 1 article^[13]^ enrolled patients with surgery, and the remaining^[14,16]^ papers included patients with multidisciplinary treatments. The cutoff value of CAR ranged from 0.04 to 0.6712. The prognostic outcomes were directly extracted from the eligible articles on OS or PFS/RFS/DFS. Univariate analysis of the HRs and 95% CIs were conducted in all studies while multivariate analysis in 5 articles. The NOS scores of the enrolled studies ranged from 6 to 8, indicating high quality.

**Table 1:** Main characteristics of all eligible studies in the meta-analysis.

Author/yr	Country	Sample size	Age (yr)	Location (colon/rectum)	Treatment	Follow-up (mo)	Cutoff value		High expression (%)	Outcome	Confounding variables	NOS score
Shibutani et al (2016) ^[11]^	Japan	99	63 (27–86)	57/42	Chemotherapy	20.8 (2.6–73.2)	0.183		36 (36.4)	OS	Gender, age, tumor location, histological type, peritoneal dissemination, no. of metastasis, CEA, molecular targeted therapy, mGPS, NLR	7
Ni et al (2016) ^[12]^	China	148	60.2 (20–74)	104/44	Chemotherapy	12 (0.4–67)	0.6712		45 (30.4)	OS	Sex, age, tumor location, neutrophils, platelets, lymphocytes, monocytes, globulin, hemoglobin, CRP	6
Solaini et al (2016) ^[13]^	UK	194	66 (59–73)	113/81	Surgery	27 (IQR 10–42)	0.133		NA	OS	Age, CRP, albumin, GPS	7
Haruki et al (2017) ^[14]^	Japan	106	64.5 (39–87)	NA	Mixed	Up to 120	0.04		59 (55.7)	OS/DFS	No. of lymph node metastases, tumor number and size, CEA, mGPS, neoadjuvant chemotherapy	8
Shibutani et al (2019) ^[15]^	Japan	40	47.5% cases > 68	40/0	Chemotherapy	Up to 36	0.122		19 (47.5)	OS/PFS	Gender, age, tumor location, no. of metastasis, RAS status, PS, no. of prior regimens, LDH, combined targeted therapy	6
Sakamoto et al (2020) ^[16]^	Japan	184	63.1 (25–94)	184/0	Mixed	Up to 41	0.093		33 (17.9)	OS/RFS	Gender, age, tumor location, tumor number, and size, CEA, CA19–9	8

CA19–9 = carbohydrate antigen 19–9, CEA = carcinoembryonic antigen, CRP = C-reactive protein, DFS = disease-free survival, GPS = Glasgow Prognostic Score, IQR = interquartile range, LDH = lactate dehydrogenase, mGPS = modified Glasgow Prognostic Score, NLR = neutrophil-lymphocyte ratio, NA = not available, NOS = Newcastle–Ottawa Quality Assessment Scale, OS = overall survival, PFS = progression-free survival, PS = performance status, RAS status = RAS type GTPase family status, RFS = recurrence-free survival.

### 3.3 Synthesis analysis

**Figure 2:**
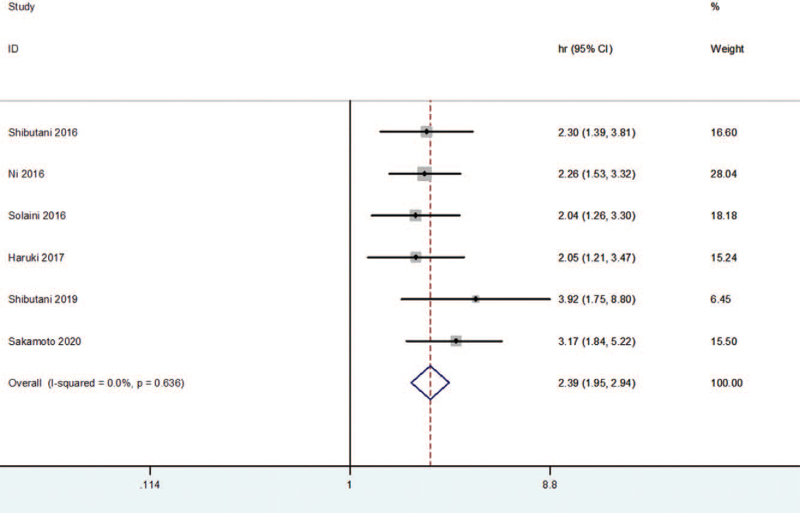
Forest plot of HR for the association between CAR and OS. CAR = C-reactive protein to albumin ratio, CI = confidence interval, HR = hazard ratio, OS = overall survival.

Six eligible studies^[11–16]^ explored the prognostic value of CAR in patients with mCRC. As shown in Figure 2, the pooled results revealed that elevated CAR was significantly associated with poorer OS (HR: 2.393; 95% CI: 1.949–2.938, *P* < .01) in a fixed-effects model. Subgroup analyses, on the basis of cutoff value of CAR, further confirmed that the relationship between high CAR and worse OS was found in patients with CAR ≥0.1 (HR: 2.324, 95% CI: 1.816–2.974, *P* < .01) and patients with CAR<0.1 (HR: 2.555, 95% CI: 1.764–3.699, *P* < .01). In addition, country, treatment, sample size, NOS score, and follow-up also did not change the predictive value of CAR in mCRC (Table 2).

**Table 2:** Pooled hazard ratios of patients’ survival according to subgroup analyses.

						Heterogeneity	
Subgroup	No. of studies	No. of patients	HR (95%CI)	Model	*P* value	*I*^2^ (%)	*P*
Overall	6	771	2.393 (1.949, 2.938)	Fixed	.000	0.0	.636
Country							
Japan	4	429	2.604 (1.968, 3.445)	Fixed	.000	0.0	.465
China	1	148	2.256 (1.531, 3.324)	–	.000	–	–
UK	1	194	2.040 (1.261, 3.301)	–	.004	–	–
Cutoff value of CAR							
≥0.1	4	481	2.324 (1.816, 2.974)	Fixed	.000	0.0	.589
<0.1	2	290	2.555 (1.764, 3.699)	Fixed	.000	24.7	.249
Treatment							
chemotherapy	3	287	2.435 (1.827, 3.245)	Fixed	.000	0.0	.464
surgery	1	194	2.040 (1.261, 3.301)	–	.004	–	–
mixed	2	290	2.555 (1.764, 3.699)	Fixed	.000	24.7	.249
Sample size							
≥150	2	378	2.499 (1.754, 3.559)	Fixed	.000	32.5	.223
<150	4	393	2.341 (1.819, 3.012)	Fixed	.000	0.0	.604
NOS score							
≥7	4	583	2.337 (1.814, 3.012)	Fixed	.000	0.0	.602
<7	2	188	2.502 (1.764, 3.549)	Fixed	.000	31.7	.226
Follow-up (mo)							
≥60	3	353	2.214 (1.698, 2.887)	Fixed	.000	0.0	.945
<60	3	418	2.687 (1.943, 3.715)	Fixed	.000	19.6	.288

CAR = C-reactive protein to albumin ratio, CI = confidence interval, HR = hazard ratio, NOS = Newcastle–Ottawa Quality Assessment Scale.

Three articles ^[14–16]^ investigated the correlation between CAR and PFS/DFS/RFS. Similar to the above results of OS, the synthesis analysis demonstrated that decreased PFS/DFS/RFS was found in patients with high CAR (HR: 1.731, 95% CI: 1.261– 2.375, *P* < .01).

A sum of 10 variables were investigated in the current meta-analysis, including age, gender, tumor location, detection of metastatic tumor, differentiation, number of organs affected by metastasis, CEA, mGPS, serum LDH level, and NLR. The pooled data indicated an obvious relationship between CAR and mGPS (2 vs 0/1; OR = 33.394, 95% CI: 12.551–91.749, *P* < .01), NLR (≥3 vs <3; OR = 2.285, 95% CI: 1.028–5.076, *P* < .05). However, no positive association was defined between CAR and age, gender, tumor location, detection of metastatic tumor, no. of organs affected by metastasis, serum LDH level, differentiation, and CEA. The details of the relationship between CAR and clinicopathological parameters are summarized in Table 3.

**Table 3:** Meta-analysis of the correlation between C-reactive protein to albumin ratio and clinicopathological characteristics of metastatic colorectal cancer.

						Heterogeneity	
Characteristics	No. of studies	No. of patients	OR (95%CI)	Model	*P* value	*I*^2^ (%)	*P*
Age (≥median vs <median)	3	387	0.669 (0.400, 1.120)	Fixed	.126	13.0	.317
Gender (male vs female)	4	393	1.194 (0.772, 1.845)	Fixed	.426	43.4	.151
Tumor location (colon vs rectum)	3	493	1.315 (0.825, 2.095)	Fixed	.249	0.0	.424
Detection of metastatic tumor (metachronous vs synchronous)	2	300	0.579 (0.326, 1.029)	Fixed	.063	0.0	.723
No. of organs affected by metastasis (multiple vs one)	3	245	1.547 (0.924, 2.589)	Fixed	.097	11.2	.324
mGPS (2 vs 0/1)	3	353	33.394 (12.551, 91.749)	Fixed	.000	0.0	.704
Serum LDH level (≥300 U/L vs <300 U/L)	1	57	2.875 (0.893, 9.258)	–	.077	–	–
NLR (≥3 vs <3)	1	129	2.285 (1.028, 5.076)	–	.043	–	–
Differentiation (poorly vs well/moderate)	1	120	0.722 (0.189, 2.755)	–	.634	–	–
CEA (≥5 ng/mL vs <5 ng/mL)	1	132	1.199 (0.363, 3.958)	–	.765	–	–

CEA = carcinoembryonic antigen, CI = confidence interval, LDH = lactate dehydrogenase, mGPS = modified Glasgow Prognostic Score, NLR = neutrophil–lymphocyte ratio, OR = odds ratio.

**Figure 3:**
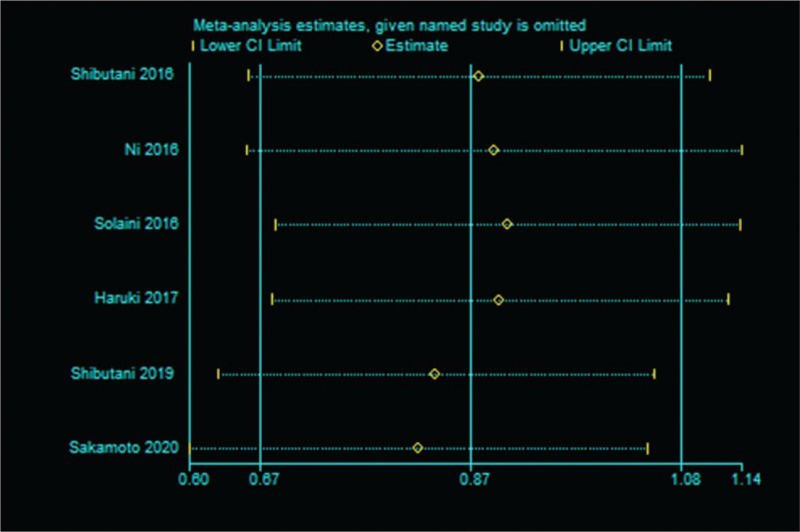
Sensitivity analysis of the relationship between CAR and OS. CAR = C-reactive protein to albumin ratio, CI = confidence interval, OS = overall survival.

**Figure 4:**
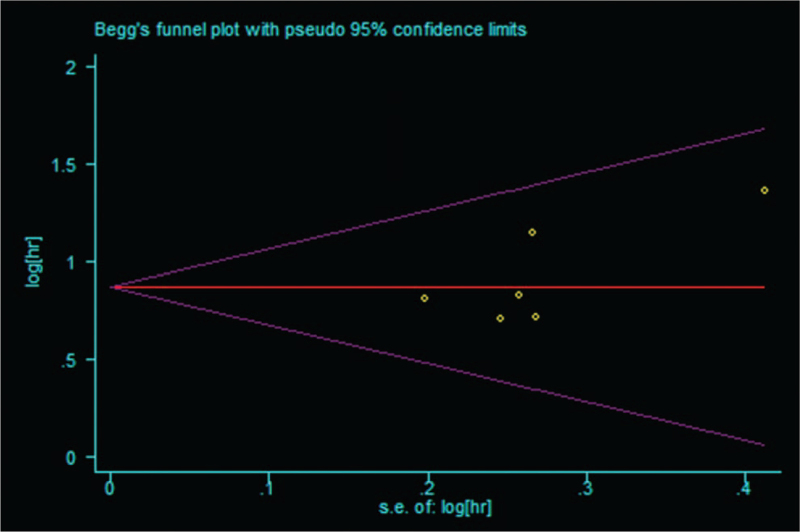
Begg funnel plot for publication bias test of OS. OS = overall survival.

Sensitivity analysis was performed to evaluate the stability of the pooled results between CAR and OS, PFS/DFS/RFS. The results revealed that slight influence was observed after removing each study, which confirmed the reliability of our conclusions (Fig. 3). No evidence of publication bias was found according to the Begg test (*P* = .260) (Fig. 4) and Egger test (*P* = .161).

## 4 Discussion

The present systematic review, including 771 patients with mCRC from 6 cohort studies,^[11–16]^ provided strong evidence of a correlation between increased CAR and poorer prognosis. The patients with elevated CAR may take higher risk of mortality than those with low CAR. Subgroup analyses further verified the stability of the prognostic value of CAR when stratified by country, cutoff value for CAR, treatment, sample size, NOS score and follow-up. In addition, elevated CAR was related to high mGPS and NLR in mCRC patients rather than other clinicopathological characteristics. Therefore, evaluation of CAR is evidently important for management of mCRC.

It is well known that inflammation plays an important role in tumorigenesis, cancer progression and metastasis through modulating tumor microenvironment.^[22]^ Cancer- related inflammation is a comprehensive release of mediator, such as cytokines, acute phase proteins, and chemokine, which promote cancer cell growth, inhibit cell apoptosis, stimulate angiogenesis, and induce chemo-resistance.^[6,23]^ Increasing evidence demonstrates that high levels of systemic inflammatory markers indicate worse prognosis in patients with CRC.^[9,10,15]^

As a novel inflammatory factor, CAR was calculated based on the serum C-reactive protein (CRP) and albumin level. CAR was initially proposed as an independent indicator for patients with sepsis^[24]^ and then was reported as a reliable survival predictor for patients with many kinds of malignancies such as esophageal cancer,^[25]^ pancreatic cancer, ^[26]^ and nasopharyngeal cancer.^[27]^

Similarly, it is reported that CAR was significantly elevated with worse survival outcome in CRC.^[28]^ Although the patients’ condition is worse in mCRC, CAR could also distinguish the patients with better prognosis by the certain cutoff value.^[14,16]^ CAR normalization after clinical treatment tended to be associated with an improved survival.^[11]^ Importantly, CAR could predict severe side effects of adjuvant chemotherapy ^[29]^ and long-term outcomes in patients with mCRC.^[14]^ However, the potential mechanisms underlying the prognostic application of CAR in mCRC remain unclear. CRP, an acute-phase protein synthesized in liver, could stimulate tumor-associated inflammatory cytokines such as TNF-α, IL-1, and IL-6, which leading to tumor deterioration.^[30]^ Researchers have found that high CRP was correlated to worse clinical outcomes of patients with mCRC.^[14]^ Meanwhile, serum level of albumin is the most common indicator of nutritional status and closely related to inflammatory response.^[31]^ Hypoalbuminemia caused by insufficient nutrient intake and excessive tumor consumption induces the activation of cytokines such as TNF-α, IL-1, and IL-6.^[32]^ Therefore, CAR can accurately reflect both the inflammatory and nutritional state and could be a potential prognostic marker for survival.

Nevertheless, several limitations in this systematic review should be interpreted. Firstly, only 6 studies with 771 patients were included in the current meta-analysis, which may generate an insufficient statistical power. Second, most of the eligible studies were conducted in Japan. Thus, the current findings should be applied with caution in Western countries. Third, all the enrolled researches were designed as retrospective cohort study, which may lead to selection bias. Fourth, the cutoff value of CAR in each study was different, which may result in inconsistent outcome threshold. Fifth, other synchronous comorbidities may influence serum CRP and albumin level.

In summary, this meta-analysis suggested that elevated CAR may serve as a promising predictive indicator in patients with mCRC. However, well-designed, large-scale prospective studies are still needed to verify these findings.

## Author contributions

**Conceptualization:** Yan Pan.

**Data curation:** Yan Pan, Yin Mei Lou, Lin Wang.

**Formal analysis:** Yan Pan, Lin Wang.

**Investigation:** Yan Pan, Yin Mei Lou, Lin Wang.

**Methodology:** Yan Pan.

**Project administration:** Yan Pan.

**Software:** Yin Mei Lou.

**Supervision:** Yan Pan.

**Validation:** Yan Pan, Yin Mei Lou, Lin Wang.

**Writing – original draft:** Yan Pan, Yin Mei Lou, Lin Wang.

**Writing – review & editing:** Yan Pan.
